# In favour of the definition “adolescents with idiopathic scoliosis”: juvenile and adolescent idiopathic scoliosis braced after ten years of age, do not show different end results

**DOI:** 10.1186/1748-7161-9-S1-O27

**Published:** 2014-12-04

**Authors:** Sabrina Donzelli, Monia Lusini, Salvatore Minnella, Fabio Zaina, Stefano Negrini

**Affiliations:** 1 ISICO - Italian Scientific Spine Institute, Milan, Italy; 2 University of Brescia - IRCCS Don Gnocchi, Brescia, Italy

## Background

The most important factor discriminating juvenile (JIS) from adolescent idiopathic scoliosis (AIS) is the risk of deformity progression. Brace treatment can change natural history, even when risk of progression is high.

## Aim

The aim of this study was to compare the end of growth results of JIS subjects, treated after 10 years of age, with final results of AIS.

## Design

Prospective observational controlled cohort study nested in a prospective database started in March 2003.

## Methods

Setting: outpatient tertiary referral clinic specialized in conservative treatment of spinal deformities.

Participants: Inclusion criteria: idiopathic scoliosis; European Risser 0-2; 25° to 45° Cobb; age 10 years or more at start of treatment. Exclusion criteria were for both groups: secondary scoliosis and pathologies known to be possible causes of scoliosis, neurological, previous treatment for scoliosis (brace or surgery).

## Groups

29 patients (26 females, 32.24±6.34 Cobb) met the inclusion criteria for AJIS, (JIS treated in adolescence), according to an x-ray before age 10. AIS group included 45 adolescents (37 females, 32.60±6.14° Cobb) with a diagnostic x-ray made after the threshold of age 10. In both groups results at the end of growth were analysed; the threshold of 5° Cobb to define worsened, improved and stabilized curves was considered. Statistical analyses: Mean and SD were used for descriptive statistics of clinical and radiographic changes. Relative Risk of progression (RR), 95% Confidence Interval (CI) of radiographic changes have been calculated.

## Results

We did not find any Cobb angle significant differences among groups at baseline and at the end of treatment. In the AJIS group the percentage of worsened was 10.3% versus 6.67% in the AIS group.

The RR of progression of AJIS was 1.35 (IC95% 0.57-3.17) versus AIS, and it wasn’t statistically significant (p=0.5338).

## Conclusion

Brace efficacy can neutralize the risk of progression. So the broad suggestion offered by these results is that there are no significant differences in the final results of AIS and JIS, treated with total respect of the SRS and SOSORT criteria, in adolescence. It is possible that JIS starting the treatment later, could be less aggressive than scoliosis that compel earlier treatment.

